# Different Methods Yielded Two-Fold Difference in Compliance with Physical Activity Guidelines on School Days

**DOI:** 10.1371/journal.pone.0152323

**Published:** 2016-03-25

**Authors:** Kerli Mooses, Jarek Mäestu, Eva-Maria Riso, Aave Hannus, Martin Mooses, Priit Kaasik, Merike Kull

**Affiliations:** Institute of Sports Sciences and Physiotherapy, Faculty of Medicine, University of Tartu, Tartu, Estonia; University of Bath, UNITED KINGDOM

## Abstract

**Introduction:**

The aim was to compare the average and the days method in exploring the compliance of children with physical activity guidelines and describe their physical activity patterns in different school day segments.

**Methods:**

Physical activity was objectively measured in 472 children aged 6–13 for one school week. Children were compliant when fulfilling PA recommendations 1) on average over all measured days (average method) or 2) on at least four measured days (days method). To explore the difference in moderate to vigorous physical activity (MVPA) minutes between compliant and non-complaint children (using both the average and days method) in various day segments, linear mixed models was used.

**Results:**

Compliance with physical activity guidelines was significantly higher with the average compared to the days method (51.7% and 23.7%, respectively). In segmented-day analysis, compliant children accrued more MVPA minutes in all day segments, especially during after-school. Gender differences appeared only during the in-school segments, where girls spent less time in MVPA (average method: -4.39 min, 95% CI = -5.36,-3.42, days method: -4.45 min, 95%CI = -5.46,-3.44). Older children accrued more MVPA minutes during physical education classes, but less during breaks, compared to younger children.

**Conclusions:**

The used methods yielded remarkably different prevalence estimates for compliance to physical activity recommendations. To ensure comparability between studies, interventions and reports, there is a need for internationally agreed operationalization and assessment methods of physical activity guidelines. As non-compliant children had lower MVPA during all day segments, greater efforts should be made to provide physical activity opportunities both during and after school.

## Introduction

To take action against increasing obesity rates and reducing physical activity levels among children, a number of governmental bodies and organisations have issued their physical activity recommendations, which suggest a minimum of 60 minutes of enjoyable and developmentally appropriate moderate to vigorous physical activity (MVPA) every day [1–4]. Numerous studies have investigated the prevalence of children compliant with the physical activity recommendations [5]. The results of these studies are often used in national reports, e.g. physical activity report cards [6–8], to assess the situation in the country and plan further actions. A problem existing in the literature is that different methods are being used to describe the proportion of children meeting the physical activity recommendations. Some studies calculate the proportion of children compliant with MVPA recommendations by evaluating the average MVPA minutes over measured days [9], e.g. dividing total time of MVPA over measured days by the number of measured days giving the average number of MVPA minutes per day, or weighed average over measured days [10–12], e.g. weighing weekend average MVPA by two and weekday average MVPA by five, while others consider children compliant when MVPA recommendations are met on every measurement day [13]. Occasionally, the approach remains unclear [14,15]. Previously, Olds et al. [16] have shown significant differences in compliance with MVPA recommendations between the most days and the four-day average method using subjectively measured physical activity data. To the best of our knowledge there is no published data comparing different methods using objectively measured physical activity data in children.

According to studies the physical activity levels of European children are alarmingly low [5]. Therefore, various intervention programmes have been implemented in different school day segments with the aim to increase daily physical activity of children [17,18]. Schools have a great potential to positively influence children’s physical activity and health behaviours regardless of the socio-economic status of every student involved. In order to develop and implement effective intervention programmes, it is essential to know which day (e.g. before-, in- or after-school) and school (e.g. academic class, break, physical education class) segments to target and in which segments MVPA minutes between children being compliant or non-compliant with physical activity recommendations differ the most. The emerging question using objectively measured data is whether the results of such analyses depend on the method (e.g. average or daily) used to classify children as compliant or non-compliant with the physical activity recommendations.

Therefore, the aim of this study was to compare the average and the days method in exploring the prevalence of 6–13 year old children compliant with WHO physical activity guidelines on school days and to describe their physical activity patterns throughout the school day.

## Methods

### Participants and Setting

Randomly chosen 13 schools throughout Estonia participated in the study conducted between December 2014 and May 2015. Participating schools had two physical education lessons per week, 10-minute breaks between classes and a 15–20 minute lunch break.

First (aged 7–9 years) and second (aged 10–13 years) school level children and their parents received written information about the study. All those willing to participate gave written informed consent (57%, n = 819). For measuring objective physical activity 636 randomly chosen children from consented children were provided an accelerometer.

The study was approved by the University of Tartu Ethics Committee (nr 244/M-11) conformed with the Declaration of Helsinki.

### Instrumentation and Procedure

Physical activity was objectively measured using ActiGraph accelerometer model GT3X (ActiGraph LLC, Penascola, FL, USA). Physical activity data was recorded every 15 second epochs. Participants were instructed to: 1) wear the accelerometer on their hip throughout the day and only remove it only for water-based activities (e.g. swimming, showering, etc.); 2) to retain their usual activity patterns. All participants kept a daily accelerometer diary, where they marked: 1) the beginning and end times of the school day and physical education classes; 2) sleep time; 3) non-wear time. On the first measurement day each child’s height (Seca 213, Seca GmbH, Germany) and body mass (A&D Instruments, Abington, UK) were measured at school by a trained researcher to the nearest 0.1 cm and 0.1 kg respectively. Body mass index (BMI) and BMI z-scores were calculated. Children were classified as underweight, normal weight, overweight or obese according to the International Obesity Task Force age-specific BMI cut-off points [19].

Data were downloaded from accelerometer and processed using ActiLife software version 6.11.2 (ActiGraph LLC, Penascola, FL, USA). Consecutive 20 minutes of zero counts were classified as non-wear time. The inclusion criteria for analysis was at least 10 hours of recorded data per day (hours awake) for a minimum of four school days [20]. Minutes of MVPA were calculated using Evenson cut-off points for children [21,22]. Time in MVPA was calculated for the whole day and for the different day segments 1) before-school (from wakening until the beginning of the school day); 2) in-school; 3) after-school (from the end of the school day until sleep or 24:00). Additionally, in-school MVPA was analysed in detail: 1) class (without physical education class); 2) physical education class; 3) break. The times for each segment were obtained by combining the information from the accelerometer diaries with school timetables.

Compliance with physical activity recommendations was assessed using two different methods:

Method 1 Average method (AM). Children were considered compliant with the physical activity recommendations when their average MVPA over all measured days was 60 minutes or more [9].
Method 2 Days method (DM). Children were considered compliant with the physical activity recommendations when they had 60 or more MVPA minutes for at least four measured days [20].

### Statistical Analysis

Descriptive statistic means, standard deviations and 95% confidence intervals were calculated. Differences in compliant and non-compliant children were assessed with chi-square and Mann-Whitney U tests. The significance level was set at p < .05.

To explore any difference in MVPA between compliant and non-complaint children (using the AM or DM) in various day segments, we used linear mixed models that enabled to take into account both the nested structure and the repeated nature of the data [23]. In our analysis we used three level models: 1) days; 2) children; 3) schools. Days, children and schools were included as separate levels to control for their possible effect on children’s physical activity. Fit of the models were evaluated with Akaike Information Criterion (AIC) and log-likelihood method. All models were adjusted for gender, school level, BMI and time spent in the segment investigated, which were treated as fixed effects. The statistical significance of the model estimates was evaluated using 95% confidence intervals.

Data was analysed with the statistical program R version 3.0.2 (http://www.r-project.org/), for linear mixed models the package lme4 was used [24,25].

## Results

From the analysis, 164 children were excluded due to: a) accelerometer malfunction (*n* = 19); b) not meeting the wear time criteria (n = 143); c) not returning the device (n = 2). Excluded children did not differ from those entered into the analysis in terms of gender, BMI and school level (p = 0.344, p = 0.197, p = 0.067 respectively).

**Fig 1:**
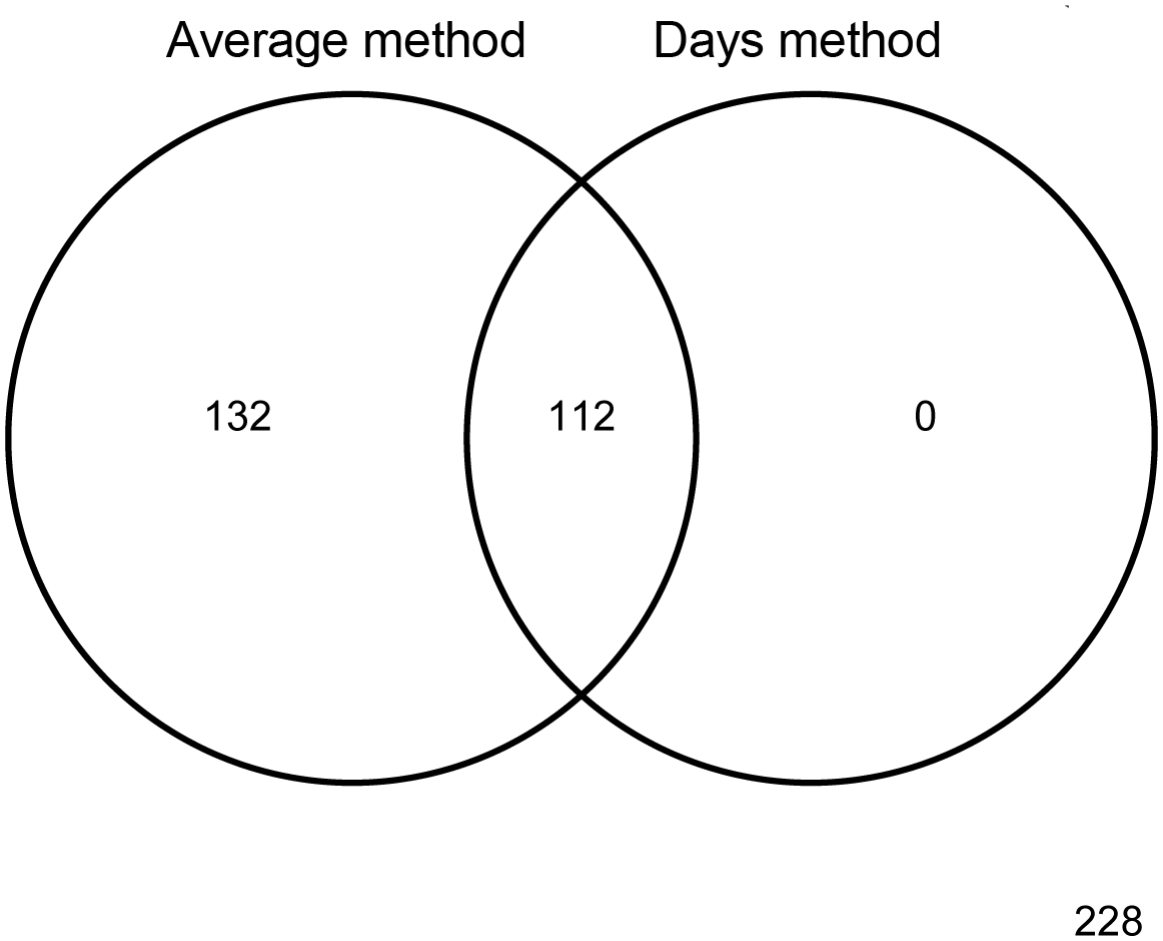
Venn diagrams showing the number of children defined as compliant or non-compliant according to average and days methods.

The prevalence of children meeting the physical activity recommendations was significantly higher with the AM compared to the DM (51.7% and 23.7%, respectively, χ^2^(1) = 137.2, p < 0.001) (Fig 1). With both methods there was no difference between children compliant and non-complaint with physical activity recommendations in terms of BMI (AM U = 21753.5, p = 0.204; DM U = 16054.5, p = 0.254) (Table 1). As for gender, there were significantly more boys than girls compliant with physical activity recommendations with both methods (AM χ^2^(1) = 4.68, p = 0.034; DM χ^2^(1) = 9.32, p = 0.002). Most MVPA minutes was accrued in after-school segment, where children compliant with physical activity recommendations spent 61.1 to 68.8 (AM and DM respectively) minutes in MVPA, whereas non-complaint children spent only 28.5 to 37.7 (AM and DM respectively) minutes in MVPA. In-school MVPA accounted for 21.8% to 22.8% (DM and AM respectively) of compliant and 27.2% to 29.2% (DM and AM respectively) of non-compliant children’s total daily MVPA on school days. Compliant children spent more than 31% of physical education class in MVPA (AM 31.3%; DM 34.2%), whereas non-compliant children less than 27% (AM 24.8%; DM 26.3%). In other in-school segments the time spent in MVPA was relatively low—compliant children spent less than 3% of class time (AM 2.4%; 2.6% DM) and approximately 15% of break time (AM 14.2%; 15.8% DM) in MVPA, whereas non-compliant children spent less than 2% of class time (AM 1.7%; 1.9% DM) and less than 10% of break time (AM 8.8%; 10.2% DM) in MVPA.

**Table 1:** Demographic characteristics of participants and their average school day physical activity with average and days method.

	Average method				Days method			
	Compliant (*n* = 244)		Non-compliant (*n* = 228)		Compliant (*n* = 112)		Non-compliant (*n* = 360)	
**Gender: Boys (%)**	52.0		42.1*		59.8		43.3*	
**School level: First level (%)**	61.1		43.9*		65.2		48.9*	
**Weight status: Under and normal weight (%)**	72.2		70.2		70.5		71.5	
	m ± SD	95% CI	m ± SD	95% CI	m ± SD	95% CI	m ± SD	95% CI
**Age (yr)**	9.1 ± 1.6	8.9, 9.3	9.6 ± 1.7	9.4, 9.8*	9.00 ± 1.6	8.6, 9.3	9.5 ± 1.7	9.3, 9.6*
**Stature (cm)**	140.0 ± 11.4	138.5, 141.5	143.2 ± 11.9	141.6, 144.8*	140.3 ± 10.3	138.3, 142.2	142.0 ± 12.2	140.7, 143.3
**Body mass (kg)**	36.1 ± 10.6	34.7, 37.5	39.3 ± 13.1	37.5, 41.1*	35.6 ± 8.9	33.9, 37.3	38.3 ± 12.8	36.9, 39.7
**BMI**	18.1 ± 3.1	17.7, 18.5	18.7 ± 3.8	18.2, 19.3	17.9 ± 2.8	17.3, 18.4	18.6 ± 3.6	18.2, 19.0
**Daily MVPA (min)**	84.7 ± 33.7	82.7, 86.7	44.7 ± 19.9	43.5, 45.9*	95.3 ± 31.2	92.5, 98.0	55.6 ± 29.4	54.2, 57.1*
**Before-school MVPA (min)**	7.0 ± 5.9	6.6, 7.4	4.1 ± 3.8	3.9, 4.4*	8.5 ± 6.8	7.9, 9.2	4.6 ± 4.1	4.4, 4.9*
**In-school MVPA (min)**	18.2 ± 12.9	17.4, 19.0	12.9 ± 10.4	12.3, 13.5*	19.9 ± 13.6	18.7, 21.1	14.3 ± 11.2	13.7, 14.8*
**Class time MVPA (min)**	5.2 ± 6.5	4.8, 5.6	3.8 ± 5.0	3.5, 4.1*	5.7 ± 7.0	5.1, 6.3	4.1 ± 5.4	4.1, 4.4*
**Break time MVPA (min)**	6.6 ± 4.7	6.3, 6.9	4.5 ± 3.6	4.3, 4.7*	7.1 ± 5.0	6.7, 7.6	5.1 ± 4.0	4.9, 5.3*
**Physical education MVPA (min)**	15.1 ± 10.0	14.1, 16.0	12.1 ± 8.3	11.3, 12.9*	16.5 ± 10.7	15.0, 17.9	12.7 ± 8.6	12.1, 13.4*
**After-school MVPA (min)**	61.1 ± 31.8	59.2, 63.0	28.5 **±** 17.1	27.5, 29.6*	68.8 ± 31.0	66.1, 71.5	37.7 ± 26.1	36.4, 39.0*

*—statistically significant difference between children compliant and non-compliant with physical activity recommendations, p < 0.05; MVPA—moderate to vigorous physical activity; BMI—body mass index

As for the multilevel analysis, the model fit according to the AIC and log-likelihood values was similar for the AM and DM. Also, both methods produced statistically similar results (Table 2). During each day segment investigated, compliant children were more engaged in MVPA than non-compliant children. The largest significant difference in MVPA minutes was during the after-school segment, with compliant children accruing 25.64 to 27.09 minutes (DM and AM respectively) more MVPA than non-compliant children. In the other day segments observed the difference was less than six minutes. Gender differences were present only during in-school segments, where overall boys spent four minutes more in MVPA than girls. As for school level, children in second school level (age 11.0 ± 0.7 years) spent two more minutes in MVPA during physical education class and approximately one minute less during breaks compared to first school level (age 7.9 ± 0.6 years).

**Table 2:** Multilevel analysis of moderate to vigorous physical activity during different school day segments with average and days methods.

		Average method			Days method		
Outcome measure	Variable	Estimate	SE	95% CI	Estimate	SE	95% CI
**Day MVPA**	**Intercept**	10.56	7.50	-4.13, 25.25	25.68	8.23	9.55, 41.82
	**Gender**^a^	-5.33	1.43	-8.14, -2.52	-5.00	1.67	-8.27, -1.73
	**School level**^b^	-3.12	1.52	-6.10, -0.14	-4.39	1.77	-7.85, -0.93
	**BMI**	-0.14	0.21	-0.56, 0.28	-0.38	0.25	-0.87, 0.10
	**Time in segment**	0.06	0.01	0.05, 0.08	0.06	0.01	0.05, 0.08
	**PA group**^c^	35.69	1.51	32.73, 38.66	33.59	1.98	29.71, 37.47
	**Total variance (%)**	11.88			23.34		
**Before-school MVPA**	**Intercept**	-2.55	1.32	-5.14, 0.03	-1.86	1.27	-4.35, 0.63
	**Gender**^a^	-0.09	0.36	-0.08, 0.62	0.05	0.36	-0.66, 0.76
	**School level**^b^	0.74	0.38	-0.01, 1.49	0.72	0.38	-0.02, 1.46
	**BMI**	0.06	0.05	-0.05, 0.16	0.05	0.05	-0.06, 0.15
	**Time in segment**	0.06	0.00	0.05, 0.07	0.06	0.00	0.05, 0.07
	**PA group**^c^	3.09	0.38	2.34, 3.83	3.69	0.43	2.86, 4.53
	**Total variance (%)**	52.63			51.64		
**In-school MVPA**	**Intercept**	8.43	2.08	4.35, 12.52	11.36	2.08	7.28, 15.44
	**Gender**^a^	-4.39	0.50	-5.36, -3.42	-4.45	0.51	-5.46, -3.44
	**School level**^b^	-0.29	0.58	-1.42, 0.83	-0.54	0.59	-1.70, 0.61
	**BMI**	-0.08	0.07	-0.23, 0.06	-0.12	0.08	-0.28, 0.02
	**Time in segment**	0.05	0.00	0.05, 0.06	0.05	0.00	0.05, 0.06
	**PA group**^c^	5.33	0.53	4.30, 6.36	4.33	0.61	3.13, 5.53
	**Total variance (%)**	8.66			9.18		
**After-school MVPA**	**Intercept**	-10.56	6.47	-23.23, 2.12	0.62	6.99	-13.09, 14.33
	**Gender**^a^	-0.99	1.33	-3.60, 1.63	-0.70	1.48	-3.61, 2.21
	**School level**^b^	-1.63	1.46	-4.49, 1.24	-2.52	1.61	-5.68, 0.64
	**BMI**	-0.09	0.20	-0.48, 0.30	-0.27	0.22	-0.07, 0.17
	**Time in segment**	0.09	0.01	0.08, 0.11	0.09	0.01	0.08, 0.11
	**PA group**^c^	27.09	1.42	24.32, 29.87	25.64	1.77	22.18, 29.10
	**Total variance (%)**	16.00			24.85		
**Break time MVPA**	**Intercept**	3.47	0.85	1.80, 5.14	4.57	0.86	2.88, 6.26
	**Gender**^a^	-1.95	0.23	-2.40, -1.50	-1.97	0.24	-2.44, -1.50
	**School level**^b^	-0.77	0.26	-1.28, -0.25	-0.86	0.27	-1.39, -0.33
	**BMI**	-0.02	0.03	-0.08, 0.04	-0.04	0.04	-0.11, 0.03
	**Time in segment**	0.11	0.01	0.10, 0.13	0.11	0.01	0.10, 0.13
	**PA group**^c^	2.01	0.24	1.54, 2.49	1.61	0.28	1.06, 2.17
	**Total variance (%)**	26.56			28.88		
**Physical education MVPA**	**Intercept**	-3.44	2.41	-8.16, 1.28	-2.77	2.38	-7.43, 1.89
	**Gender**^a^	-2.02	0.59	-3.17, -0.87	-1.89	0.60	-3.06, -0.73
	**School level**^b^	2.00	0.62	0.77, 3.22	1.91	0.63	0.68, 3.13
	**BMI**	-0.13	0.09	-0.30, 0.04	-0.14	0.09	-0.31, 0.03
	**Time in segment**	0.38	0.03	0.33, 0.43	0.38	0.03	0.33, 0.43
	**PA group**^c^	2.65	0.61	1.45, 3.86	2.87	0.69	1.52, 4.21
	**Total variance (%)**	20.29			21.09		
**Class MVPA**	**Intercept**	2.74	1.12	0.54, 4.94	3.81	1.11	1.64, 5.98
	**Gender**^a^	-1.00	0.27	-1.54, -0.47	-1.03	0.28	-1.58, -0.49
	**School level**^b^	-0.43	0.30	-1.03, 0.16	-0.52	0.31	-1.13, 0.09
	**BMI**	0.01	0.04	-0.07, 0.09	-0.01	0.04	-0.09, 0.08
	**Time in segment**	0.02	0.00	0.01, 0.02	0.02	0.00	0.01, 0.02
	**PA group**^c^	1.78	0.29	1.21, 2.34	1.36	0.33	0.70, 2.01
	**Total variance (%)**	14.30			14.45		

^a^Reference category: Boys.

^b^Reference category: First school level.

^c^Reference category: Non-compliant.

MVPA—moderate to vigorous physical activity; BMI—body mass index; PA group—physical activity group.

## Discussion

The current study compared the average (average MVPA over measured days, AM) and days method (MVPA on every measured day, DM) in categorising children as compliant or non-compliant with physical activity recommendations on school days and in the analysis of day (before-, in- and after-school) and school (academic class, break, physical education class) segments. To the best of our knowledge our study is the first to compare these two methods using objectively measured physical activity data in children. One main finding was that using objectively measured physical activity data, the AM gave a significantly higher prevalence of children compliant to the physical activity recommendations than did the DM. The prevalence estimates of children compliant with the physical activity recommendations was 24% and 52% with the DM and AM respectively, which is a smaller difference than previously found with subjectively measured physical activity data, where threefold difference between average and all days methods was present [16]. Still, the difference between the AM and DM used in our study was remarkable. Therefore, we agree with Olds et al. [16] that results from studies using different methods are not comparable.

An approach where children have to meet physical activity recommendations on all days without exception seems to best correspond with current WHO physical activity guidelines which state that children should engage in 60 minutes of MVPA daily [4]. However, the limitation of every day method is that due to external factors (e.g. travelling, sickness, weather conditions etc.) children might fail to meet the recommendations on all measured days and are therefore classified as non-compliant, even when they have met the recommendation on all other days. In the present study, to be classified as compliant by the DM, children had to engage in 60 minutes of MVPA on at least four days out of five. In a systematic review, Janssen and LeBlanc [26] pointed out that to maintain good health, at least 60 minutes of MVPA every day might not be needed and therefore they suggested that an AM would be suitable for calculating the prevalence of children meeting the physical activity guidelines. Moreover, the strength of the AM was that children were classified as compliant with the recommendations even if on some days they were a few minutes below the norm but fulfilled the norm on the other days. We would argue that there is not much difference in health outcomes whether child spends a 58 or 62 minutes in MVPA during a day [25], but when strictly following WHO recommendations for classification such child would be classified as non-compliant. Another key point is that although it has been shown that the reliability of 4 days of measurement is acceptable [20], there is lack of studies exploring the relation between the number of days measured and the prevalence of children meeting the physical activity guidelines. For example, it is more likely to comply with the guidelines on four days rather than over a week or two. Accurate and reliable assessment of children meeting or not meeting physical activity recommendations is extremely important for reporting the current situation in different populations, comparing results between studies, finding physically inactivity risk groups and planning interventions. In the present study based on a data of one school week, the methods used to estimate the prevalence of children meeting the physical activity recommendations resulted in a two-fold difference. Therefore, the standardisation of physical activity guideline operationalization and assessment method is urgently needed in order to have comparable results between different studies.

Another important outcome of our study was that differences in MVPA minutes between compliant and non-compliant children during different day segments was similar between the AM and the DM models. Children compliant with the WHO physical activity recommendations accrued more MVPA minutes in all the day segments investigated compared to non-compliant children. In our study, the biggest difference in MVPA minutes between compliant and non-compliant children was observed during the after-school segment, which is in line with previous objectively and subjectively measured data [27,28]. Unfortunately, we had no information about the way children commuted to and from school, hence we could not assess the possible effect of active commuting on daily MVPA. Previous studies suggest that active transport can contribute to overall physical activity [29–31]. In addition, it has been suggested that the biggest differences in physical activity appear after school between 4 pm and 6.30 pm which is the time when physical activity and screen time compete [27,32,33]. Thus, a more detailed analysis of the after-school segment, including any participation in organised sport, warrants further investigation. Since the after-school segment had the largest difference in MVPA minutes between compliant and non-compliant children, more attention should be paid to creating leisure time physical activity opportunities that appeal to children who are currently less active after school.

Although the magnitude of the difference during the in-school segment was smaller than that of the after-school segment, compliant children still accrued more MVPA minutes during the in-school segment compared to non-compliant children, which confirms previous findings [28]. Differences between compliant and non-compliant children were present in all school segments explored, with greatest differences in physical education lesson. From gender perspective, our findings confirmed that boys spend significantly more time in MVPA during breaks between classes compared to girls [34,35]. At the same time, gender differences were only present during in-school segment and not before- or after-school segments, which could suggest an inequality of physical activity opportunities in school setting. We speculate that our results were due to current school rules that—mostly for safety reasons—prohibit running and girls may be more prone to follow such rule than boys, as it has been found that girls have higher self-discipline [36], however further research is needed. Nonetheless, schools should focus on creating an environment that supports and favours the physical activity of all children throughout the school day, as MVPA during breaks and lessons was low (below 15% and 3% respectively).

Considering school level, another interesting finding emerges from our study—children from second school level (age 11.0 ± 0.7 years) spent more time in MVPA during physical education classes compared to first school level (age 7.9 ± 0.6 years) children. This result is in contrast with the overall tendency that both daily and in-school physical activity levels decrease with age [10,37]. We hypothesize that one possible reason was the quality of physical education class, as physical education classes at first school level were organised by class teachers, whereas at second school level by specially trained physical education teachers. It has been shown that specialist-taught physical education lesson can significantly increase the time spent in MVPA [38,39]. Although the proportion of MVPA during physical education class was higher in second school level (age 11.0 ± 0.7 years) compared to first school level (age 7.9 ± 0.6 years), students still spent a relatively low proportion (less than 34%) of their physical education classes in MVPA indicating that more attention should be paid on the quality of physical education class.

Overall, the school-related physical activity contributed considerably to children’s daily physical activity as more than 22% of daily MVPA minutes were accrued at school, which is in line with previous findings [37,40]. Still, more recent studies have shown that school time MVPA can account for more than 40% of daily MVPA [41,42] showing the great potential of in-school segment in supporting children physical activity.

When interpreting the results we have to consider that although ActiGraph accelerometers have shown to be valid to measure physical activity of children [21], there are some activities that are not well detected (e.g. cycling, swimming) and therefore physical activity level in current study can be underestimated. We also did not control for socio-economic status or parental education, which may help to gain better understanding of the influence of these factors on physical activity of children. Although we controlled for BMI, there is no information about maturity, which could give more insight whether maturity affects gender differences in MVPA [43,44]. In addition, when describing the overall physical activity levels whole week data should be used as physical activity during weekends tends to be lower than on school days [45]. However, as the aim of current study was to explore the physical activity on school days and we used conservative inclusion criteria by including only children who had accelerometer data for at least four days out of five, we can consider it the strength of our study. Likewise, we were able to reduce the potential misclassification of day segments by combining information about school attendance, physical education classes and break times from the children’s self-report diaries and the school timetables.

## Conclusions

Caution must be used when comparing prevalence estimates of compliance to physical activity recommendations from different studies, because contradictory and incomparable results can be obtained when using the AM or DM to calculate the prevalence of children meeting physical activity guidelines. To ensure comparability between studies, interventions and reports, there is a need for internationally-agreed operationalization and assessment methods.

The current study revealed that children who met the physical activity recommendations were more active during all day segments than those who did not. It is reasonable to conclude that interventions and public health programmes should target both the after-school segment, where the differences in MVPA minutes between children compliant and non-compliant with physical activity recommendations were largest, as well as the in-school segment, where the focus should be on creating an environment which supports the physical activity among all children during the lessons, breaks and physical education lesson.
